# First record of *Aximopsis* s.l. (Hymenoptera, Eurytomidae) from China, with description of a new species parasitising the larvae of the coastal pollinator *Ceratina smaragdula* (Hymenoptera, Apidae)

**DOI:** 10.3897/BDJ.14.e206796

**Published:** 2026-09-25

**Authors:** Zixuan Li, Y. Miles Zhang, Zhixuan Liu, Shixiao Luo, Huayan Chen

**Affiliations:** 1 State Key Laboratory of Plant Diversity and Specialty Crops and Laboratory of Plant Resources Conservation and Sustainable Utilization, South China Botanical Garden, Chinese Academy of Sciences, Guangzhou, China State Key Laboratory of Plant Diversity and Specialty Crops and Laboratory of Plant Resources Conservation and Sustainable Utilization, South China Botanical Garden, Chinese Academy of Sciences Guangzhou China; 2 South China National Botanical Garden, Guangzhou, China South China National Botanical Garden Guangzhou China; 3 University of Chinese Academy of Sciences, Guangzhou, China University of Chinese Academy of Sciences Guangzhou China; 4 Institute of Ecology and Evolution, University of Edinburgh, Edinburgh, United Kingdom Institute of Ecology and Evolution, University of Edinburgh Edinburgh United Kingdom

**Keywords:** Eurytominae, *nodularis* group, parasitoid, small carpenter bee

## Abstract

**Background:**

The genus *Aximopsis* *sensu lato* (Hymenoptera, Eurytomidae) is a cosmopolitan group of parasitoid wasps, currently comprising 43 described species. The genus is defined by a suite of homoplastic morphological characters and its taxonomic boundaries remain unstable, with recent molecular phylogenies indicating paraphyly. The *nodularis* species group, which accounts for approximately half of the known species, includes parasitoids of various endophytic insects. While host associations with small carpenter bees of the genus *Ceratina* (Apidae) have been documented for a few eurytomid species in Europe and North America, no species of *Aximopsis* s.l. has ever been recorded from China.

**New information:**

A new species of *Aximopsis* Ashmead, *Aximopsis qiong* Li, Zhang & Chen sp. nov. (Hymenoptera, Chalcidoidea, Eurytomidae) is described from Hainan, China. The species was reared as a gregarious ectoparasitoid of the small carpenter bee *Ceratina smaragdula* (Fabricius) (Hymenoptera, Apidae). The new species is provisionally placed in **Aximopsis* *sensu lato** and tentatively assigned to the *nodularis* species group based on morphological characters. Diagnostic characters, detailed morphology, biological observations and illustrations of both sexes are provided. Molecular phylogenetic analysis, based on *COI* sequences,failed to recover **Aximopsis* *sensu lato** as monophyletic, supporting previous evidence of its uncertain generic boundaries. This discovery represents the first record of the genus *Aximopsis* from China and only the second confirmed species of the *nodularis* group associated with *Ceratina* hosts. Collectively, our finding expand the known distribution and host range of this parasitoid lineage, while also underscoring the pressing need for a comprehensive taxonomic revision of **Aximopsis* *sensu lato**.

## Introduction

The genus **Aximopsis* *sensu lato** Ashmead, 1904 (Eurytomidae) was re-defined by Lotfalizadeh et al. (2007) to include *Conoaxima* Brues, 1922, *Eurytomaria* Masi, 1943, *Mesoeurytoma* Cameron, 1911, *Aximogastroma* Narendran, 1994 and the *Eurytoma nodularis* Boheman, 1836 species group. This genus is currently recognised, based on a suite of homoplastic morphological characters: postgenal laminae raised, prepectus with a strong medioventral tooth, subventral carinae of prepectus delimiting a raised surface, subventral prepectus with a deep pit, mesopleuron with a horizontal ventral shelf and a completely delimited epicnemium (Lotfalizadeh et al. 2007, Gates et al. 2025). The 43 described species of **Aximopsis* s.l*.. are found worldwide, except in Australia and Antarctica (UCD Community 2023) and the genus includes many undescribed species in tropical regions. *Aximopsis* are parasitoids of endophytic insects living in branches, stems or leaf mines; most known species are parasitoids of Coleoptera, Lepidoptera and Hymenoptera (Zerova and Seryogina 2006, Lotfalizadeh et al. 2006, Lotfalizadeh et al. 2007, Gates and Delvare 2008, Gates 2009, Lotfalizadeh and Hosseini 2014, Delvare et al. 2019, Naghizadeh et al. 2019, Lotfalizadeh et al. 2020, UCD Community 2023).

About half of the known *Aximopsis* s. l. species belongs to the *nodularis* group, which can be recognised by the presence of: 1) a conspicuous mesopleuron ventral shelf; 2) carinate fore coxae; 3) distinctly petiolate female metasoma; and 4) male funicular segments with two convex apices and covered with long pubescence (Lotfalizadeh et al. 2007, Lotfalizadeh and Hosseini 2014). The nominal species *Aximopsis nodularis* is a European species with a wide host range that includes Apidae (including *Ceratina* Latreille, 1802), Curculionidae, Cynipidae, Ichneumonidae, Sphecidae and Vespidae (UCD Community 2023). Other members of the *nodularis* group have been recorded as parasitoids of Coleoptera (Anthribidae, Buprestidae, Chrysomelidae (Bruchinae)), Diptera (Tephritidae), Hymenoptera (Apidae, Cynipidae) and Lepidoptera (Coleophoridae, Noctuidae, Pyralidae) in the Afrotropical, Neotropical and Palearctic Regions (UCD Community 2023). The placement of this group is unstable: as most were originally described within *Eurytoma* Illiger, 1807 and subsequently moved to *Aximopsis* (Lotfalizadeh et al. 2006, Zerova and Seryogina 2006, Lotfalizadeh et al. 2007, Gates and Delvare 2008, Gates 2009, Lotfalizadeh and Hosseini 2014, Delvare et al. 2019, Naghizadeh et al. 2019, Lotfalizadeh et al. 2020, UCD Community 2023). Recent phylogenomic analyses have recovered a paraphyletic relationship between **Aximopsis* s.l*.. and genera, such as *Chryseida* Spinola, 1840 and *Axima* Walker, 1862 (Zhang et al. 2025, Park et al. 2026); thus, the placement of the species group will likely change in light of revision (G. Delvare, pers. comm.).

Species from multiple genera of eurytomines have been recorded in association with *Ceratina*, including *Axima zabriskiei* Howard, 1890 on *C. dupla* Say, 1837 and *C. calcarata* Robertson, 1900 in North America (Vickruck et al. 2010); *Aximopsis nodularis* on *C. callosa* (Fabricius, 1794) in Europe (Zerova and Seryogina 2006); and *Eurytoma apiculae* Bugbee, 1966 on *C. dallatorreana* Friese, 1896, *C. nanula* Cockerell, 1897, *C. punctigena* Cockerell, 1916 and possibly *C. calcarata* in North America (Bugbee 1966, Vickruck et al. 2010). Additionally, records of undescribed *Eurytoma* species have been documented on *C. australensis* (Perkins, 1912) in Australia (Vickruck et al. 2010).

Here, we describe a new species of eurytomine reared from the larvae of *Ceratina smaragdula* (Fabricius, 1787) from Hainan Province of China that belong to the *nodularis* group and we tentatively place it within **Aximopsis* s.l*..

## Materials and methods

### Insect collection

The host bee *Ceratina smaragdula* naturally utilises dead twigs of *Scaevola taccada* (Asterales, Goodeniaceae) for nesting. To obtain nests of this host and thereby rear its parasitoid, we collected nest‑bearing twigs from two sources: (1) naturally occurring dead twigs along roadsides with visible nesting signs (a circular entrance hole and accumulated wood dust) and (2) artificial trap nests made of sun‑dried twigs placed in PVC holders near *S. taccada* thickets. Nest collection was conducted in Wenchang Town (19°33'N, 110°46'E) of Wenchang City and Yongxing Island (16°50'N, 112°20'E) of Sansha City, Hainan Province from September 2023 to December 2025, covering the peak nesting season of the host bees. All occupied twigs were gathered at dusk and sealed with tape to prevent adult insect escape.

In the laboratory, each twig was dissected longitudinally under a Nikon SMZ25 stereomicroscope. Brood cells containing parasitoid larvae or pupae were kept individually in ventilated tubes at room temperature and checked daily for adult emergence. The last parasitoids emerged in June 2026 in the laboratory. Emerged adults of parasitoids were preserved in 100% ethanol for morphological and molecular studies. All nest tubes containing insect brood (including parasitoid larvae, pupae or host remains) were preserved in 100% ethanol for further examination.

Specimens of *Aximopsis qiong* sp. nov. are deposited in two institutions: South China Botanical Garden, Chinese Academy of Sciences, Guangzhou, China (SCBG); and Institute of Zoology, Chinese Academy of Sciences, Beijing, China (IZCAS).

### Morphological terminology and abbreviations

Terminologies follows Burks et al. (2025) for general chalcidoid morphology and Lotfalizadeh et al. (2007) for eurytomid-specific terms. The following measurements and abbreviations were used: F1–Fn, the first and the following flagellomeres; POL (post-ocellar distance), the distance between the inner margins of the posterior ocelli; OOL (ocellar–ocular distance), the distance from the outer margin of lateral ocellus to the inner margin of compound eye; LOL (lateral–ocular distance), the distance between lateral and frontal ocelli; and Gt1–Gtn, the first and the following gastral tergites.

### DNA sequencing and molecular analyses

Genomic DNA was extracted from the right fore-leg of four specimens using the TIANamp Micro DNA Kit (Tiangen Biotech, Beijing, China) following the protocol of Taekul et al. (2013). The mitochondrial cytochrome oxidase subunit 1 (*COI*) gene sequences were amplified as described in Chen et al. (2023).

For phylogenetic analysis, *Phylloxeroxenus* sp. Ashmead, 1888 (GenBank accession: PV031844) was selected as the outgroup. In addition, *COI* sequences of related taxa (Delvare et al. 2019, Zhang et al. 2025) were downloaded from GenBank, including *Chryseida bennetti* Burks, 1956 (PV031771), *Chryseida burksi* Zerova, 1980 (PV031772), *Chryseida* sp6 (PV031775), *Aximopsis* sp1 (PV031741), *Aximopsis* sp2 (PV031742), *Aximopsis* sp. (MH878925), *Aximopsis gabrielae* Zhang, Gates & Campos, 2022 (PV031740), *Aximopsis balajasi* Delvare, 2019 (MH878922, PV031738) and *Aximopsis collina* (Zerova, 1984) (MH878923, PV031739). In total, 16 *COI* sequences, including the four obtained from the Chinese specimens (GenBank Accessions PZ568852-PZ568855), were used for phylogenetic analysis. The phylogenetic tree was reconstructed using IQ-TREE v.3.1.2 (Wong et al. 2026) under a Maximum Likelihood framework, using ModelFinder (Kalyaanamoorthy et al. 2017) to find the best evolutionary model. To assess support, we performed 1000 Ultrafast Bootstrap (UFBoot, Hoang et al. (2017)), along with ‘-bnni’ to reduce the risk of overestimation. The resulting tree was visualised with iTOL v.7 (Letunic and Bork 2021) and further refined in Adobe Illustrator 2025.

### Imaging

Multifocal images of voucher specimens deposited in SCBG were produced using a Nikon SMZ25 microscope, equipped with a Nikon DS-Ri 2 digital camera system (Melville, NY, USA). Photographs of live insects were taken with a Canon 5D Mark IV camera (Tokyo, Japan) fitted with a 100 mm macro lens. Image plates were subsequently assembled and post-processed using Adobe Photoshop CS6 Extended.

## Taxon treatments

### Aximopsis qiong

Li, Zhang & Chen sp. nov.

B28C88EA-21F5-5B2C-B364-BFD3F20F97A2

8D5C7682-0920-4AC6-9D4D-766A7139B5C1

#### Materials

- **Type status:** Holotype. **Occurrence:** catalogNumber: E0016503; recordedBy: Zhixuan Liu; individualCount: 1; sex: female; occurrenceID: E52C575C-83C4-5707-8BF5-1D56FC052FE7; **Taxon:** scientificName: Aximopsis qiong; vernacularName: 海南拟盔广肩小蜂; taxonomicStatus: sp. nov.; **Location:** country: China; stateProvince: Hainan; municipality: Wenchang City; locality: Wenchang Town; verbatimCoordinates: 19°33'N 110°46'E; **Event:** eventDate: 08-2025; fieldNotes: ex. Nests of Ceratina smaragdula in stems of Scaevola taccada; **Record Level:** institutionCode: SCBG; **Material Entity:** preparations: in card
- **Type status:** Paratype. **Occurrence:** catalogNumber: E0015707; recordedBy: Zhixuan Liu; individualCount: 1; sex: female; occurrenceID: 41D288C3-2DAA-5F3D-B102-4C540102DA14; **Taxon:** scientificName: Aximopsis qiong; vernacularName: 海南拟盔广肩小蜂; taxonomicStatus: sp. nov.; **Location:** country: China; stateProvince: Hainan; municipality: Wenchang City; locality: Wenchang Town; verbatimCoordinates: 19°33'N 110°46'E; **Event:** eventDate: 08-2025; fieldNotes: ex. Nests of Ceratina smaragdula in stems of Scaevola taccada; **Record Level:** institutionCode: SCBG; **Material Entity:** preparations: in card
- **Type status:** Paratype. **Occurrence:** catalogNumber: E0015709; recordedBy: Zhixuan Liu; individualCount: 1; sex: female; occurrenceID: 589B73F6-9B76-5194-91C4-9F6229EB9FF8; **Taxon:** scientificName: Aximopsis qiong; vernacularName: 海南拟盔广肩小蜂; taxonomicStatus: sp. nov.; **Location:** country: China; stateProvince: Hainan; municipality: Wenchang City; locality: Wenchang Town; verbatimCoordinates: 19°33'N 110°46'E; **Event:** eventDate: 08-2025; fieldNotes: ex. Nests of Ceratina smaragdula in stems of Scaevola taccada; **Record Level:** institutionCode: SCBG; **Material Entity:** preparations: in card
- **Type status:** Paratype. **Occurrence:** catalogNumber: E0015706; recordedBy: Zhixuan Liu; individualCount: 1; sex: male; occurrenceID: A09841FF-8D47-5B00-9620-2DC8A6ACC55B; **Taxon:** scientificName: Aximopsis qiong; vernacularName: 海南拟盔广肩小蜂; taxonomicStatus: sp. nov.; **Location:** country: China; stateProvince: Hainan; municipality: Wenchang City; locality: Wenchang Town; verbatimCoordinates: 19°33'N 110°46'E; **Event:** eventDate: 08-2025; fieldNotes: ex. Nests of Ceratina smaragdula in stems of Scaevola taccada; **Record Level:** institutionCode: SCBG; **Material Entity:** preparations: in card
- **Type status:** Paratype. **Occurrence:** catalogNumber: E0015708; recordedBy: Zhixuan Liu; individualCount: 1; sex: male; occurrenceID: 91D631A8-0A58-5EFE-A490-4FFA07987394; **Taxon:** scientificName: Aximopsis qiong; vernacularName: 海南拟盔广肩小蜂; taxonomicStatus: sp. nov.; **Location:** country: China; stateProvince: Hainan; municipality: Wenchang City; locality: Wenchang Town; verbatimCoordinates: 19°33'N 110°46'E; **Event:** eventDate: 08-2025; fieldNotes: ex. Nests of Ceratina smaragdula in stems of Scaevola taccada; **Record Level:** institutionCode: SCBG; **Material Entity:** preparations: in card
- **Type status:** Paratype. **Occurrence:** catalogNumber: E0015992; recordedBy: Zhixuan Liu; individualCount: 1; sex: female; occurrenceID: 4CCC9D25-9F3C-594A-8098-F4BFF20D91B2; **Taxon:** scientificName: Aximopsis qiong; vernacularName: 海南拟盔广肩小蜂; taxonomicStatus: sp. nov.; **Location:** country: China; stateProvince: Hainan; municipality: Wenchang City; locality: Wenchang Town; verbatimCoordinates: 19°33'N 110°46'E; **Event:** eventDate: 08-2025; fieldNotes: ex. Nests of Ceratina smaragdula in stems of Scaevola taccada; **Record Level:** institutionCode: IZCAS; **Material Entity:** preparations: in ethanol
- **Type status:** Paratype. **Occurrence:** catalogNumber: E0015991; recordedBy: Zhixuan Liu; individualCount: 1; sex: male; occurrenceID: 4F6858E9-1126-5584-BCA4-B55AFA0A7F90; **Taxon:** scientificName: Aximopsis qiong; vernacularName: 海南拟盔广肩小蜂; taxonomicStatus: sp. nov.; **Location:** country: China; stateProvince: Hainan; municipality: Wenchang City; locality: Wenchang Town; verbatimCoordinates: 19°33'N 110°46'E; **Event:** eventDate: 08-2025; fieldNotes: ex. Nests of Ceratina smaragdula in stems of Scaevola taccada; **Record Level:** institutionCode: IZCAS; **Material Entity:** preparations: in ethanol
- **Type status:** Paratype. **Occurrence:** catalogNumber: E0015993; recordedBy: Ruichong Wang; individualCount: 1; sex: female; occurrenceID: E8928A34-7C6D-58FF-B1EE-7495394ED754; **Taxon:** scientificName: Aximopsis qiong; vernacularName: 海南拟盔广肩小蜂; taxonomicStatus: sp. nov.; **Location:** country: China; stateProvince: Hainan; municipality: Sansha City; locality: Yongxing Island; verbatimCoordinates: 16°50'N 112°20'E; **Event:** eventDate: 06-2026; fieldNotes: ex. Nests of Ceratina smaragdula in stems of Scaevola taccada; **Record Level:** institutionCode: IZCAS; **Material Entity:** preparations: in ethanol
- **Type status:** Paratype. **Occurrence:** catalogNumber: E0015994; recordedBy: Ruichong Wang; individualCount: 1; sex: female; occurrenceID: 936CC93F-CF48-5DA1-B0A5-FDB91B61A5B2; **Taxon:** scientificName: Aximopsis qiong; vernacularName: 海南拟盔广肩小蜂; taxonomicStatus: sp. nov.; **Location:** country: China; stateProvince: Hainan; municipality: Sansha City; locality: Yongxing Island; verbatimCoordinates: 16°50'N 112°20'E; **Event:** eventDate: 06-2026; fieldNotes: ex. Nests of Ceratina smaragdula in stems of Scaevola taccada; **Record Level:** institutionCode: IZCAS; **Material Entity:** preparations: in ethanol
- **Type status:** Paratype. **Occurrence:** catalogNumber: E0015995; recordedBy: Ruichong Wang; individualCount: 1; sex: female; occurrenceID: 7A56745E-F92B-58D2-8857-7031E6325A86; **Taxon:** scientificName: Aximopsis qiong; vernacularName: 海南拟盔广肩小蜂; taxonomicStatus: sp. nov.; **Location:** country: China; stateProvince: Hainan; municipality: Sansha City; locality: Yongxing Island; verbatimCoordinates: 16°50'N 112°20'E; **Event:** eventDate: 06-2026; fieldNotes: ex. Nests of Ceratina smaragdula in stems of Scaevola taccada; **Record Level:** institutionCode: IZCAS; **Material Entity:** preparations: in ethanol
- **Type status:** Paratype. **Occurrence:** catalogNumber: E0015996; recordedBy: Ruichong Wang; individualCount: 1; sex: male; occurrenceID: 27E0A0A9-A70A-59AC-8660-DFF7F29F0CDA; **Taxon:** scientificName: Aximopsis qiong; vernacularName: 海南拟盔广肩小蜂; taxonomicStatus: sp. nov.; **Location:** country: China; stateProvince: Hainan; municipality: Sansha City; locality: Yongxing Island; verbatimCoordinates: 16°50'N 112°20'E; **Event:** eventDate: 06-2026; fieldNotes: ex. Nests of Ceratina smaragdula in stems of Scaevola taccada; **Record Level:** institutionCode: IZCAS; **Material Entity:** preparations: in ethanol
- **Type status:** Paratype. **Occurrence:** catalogNumber: E0015997; recordedBy: Ruichong Wang; individualCount: 1; sex: male; occurrenceID: 8D1CB5FA-624D-5B45-9944-79EB98E6CF4E; **Taxon:** scientificName: Aximopsis qiong; vernacularName: 海南拟盔广肩小蜂; taxonomicStatus: sp. nov.; **Location:** country: China; stateProvince: Hainan; municipality: Sansha City; locality: Yongxing Island; verbatimCoordinates: 16°50'N 112°20'E; **Event:** eventDate: 06-2026; fieldNotes: ex. Nests of Ceratina smaragdula in stems of Scaevola taccada; **Record Level:** institutionCode: IZCAS; **Material Entity:** preparations: in ethanol
- **Type status:** Other material. **Occurrence:** recordedBy: Zhixuan Liu; individualCount: 67; sex: female; occurrenceID: E01BBFE7-E451-5B39-B00E-F48687D36C88; **Taxon:** scientificName: Aximopsis qiong; vernacularName: 海南拟盔广肩小蜂; taxonomicStatus: sp. nov.; **Location:** country: China; stateProvince: Hainan; municipality: Wenchang City; locality: Wenchang Town; verbatimCoordinates: 19°33'N 110°46'E; **Event:** eventDate: 08-2025; fieldNotes: ex. Nests of Ceratina smaragdula in stems of Scaevola taccada; **Record Level:** institutionCode: SCBG; **Material Entity:** preparations: in ethanol
- **Type status:** Other material. **Occurrence:** recordedBy: Zhixuan Liu; individualCount: 18; sex: male; occurrenceID: B7CD0AEC-534B-5BD1-9593-13FAC28F6539; **Taxon:** scientificName: Aximopsis qiong; vernacularName: 海南拟盔广肩小蜂; taxonomicStatus: sp. nov.; **Location:** country: China; stateProvince: Hainan; municipality: Wenchang City; locality: Wenchang Town; verbatimCoordinates: 19°33'N 110°46'E; **Event:** eventDate: 08-2025; fieldNotes: ex. Nests of Ceratina smaragdula in stems of Scaevola taccada; **Record Level:** institutionCode: SCBG; **Material Entity:** preparations: in ethanol
- **Type status:** Other material. **Occurrence:** recordedBy: Ruichong Wang; individualCount: 13; sex: female; occurrenceID: E344433B-A64C-52BC-BAAB-4AE9857AA259; **Taxon:** scientificName: Aximopsis qiong; vernacularName: 海南拟盔广肩小蜂; taxonomicStatus: sp. nov.; **Location:** country: China; stateProvince: Hainan; municipality: Sansha City; locality: Yongxing Island; verbatimCoordinates: 16°50'N 112°20'E; **Event:** eventDate: 06-2026; fieldNotes: ex. Nests of Ceratina smaragdula in stems of Scaevola taccada; **Record Level:** institutionCode: SCBG; **Material Entity:** preparations: in ethanol
- **Type status:** Other material. **Occurrence:** recordedBy: Ruichong Wang; individualCount: 6; sex: male; occurrenceID: 160BD56E-5E52-5F84-BBB6-2597334ECBAB; **Taxon:** scientificName: Aximopsis qiong; vernacularName: 海南拟盔广肩小蜂; taxonomicStatus: sp. nov.; **Location:** country: China; stateProvince: Hainan; municipality: Sansha City; locality: Yongxing Island; verbatimCoordinates: 16°50'N 112°20'E; **Event:** eventDate: 06-2026; fieldNotes: ex. Nests of Ceratina smaragdula in stems of Scaevola taccada; **Record Level:** institutionCode: SCBG; **Material Entity:** preparations: in ethanol

#### Description

**Female holotype**. Body size 5.1 mm. Body (including tegula) black (Fig. 1A and B); antenna with scape, pedicel and first funicular yellow, remaining funiculars dark; legs with coxae black, femora and tibiae yellow with median area infuscate, protarsi yellow, meso‑ and metatarsi white; wing veins brown; setation of body and membrane of wings white; mandible brown on disc; exserted part of ovipositor sheaths entirely dark. Lateroventral parts of gastral tergites T2 to T4 yellow.

Head 2.5× as wide as long in dorsal view; eyes separated by 2.0× their own height; POL almost 2.1× OOL and OOL 2.1× posterior ocellus diameter; temple 0.2× as long as eye length (Fig. 2C); anterior outline of frons not obviously convex, frons with faint pre-orbital ridge; scrobal depression smooth, completely carinate laterally and dorsally, separated from anterior ocellus by a distance 0.2× its own diameter (Fig. 2B). Head 1.6× as wide as high in frontal view; frons and face with lanceolate setae, the setation orientated downwards on face, outwards on the punctured frons. Malar space 0.6× as long as height of eye and 0.9× width of oral fossa. Lower edge of toruli closer to ventral margin of clypeus than to anterior ocellus (ratio of distances: to clypeus / to ocellus = 0.7). Mandible 3‑toothed with a discal whorl of three setae. Clypeus smooth, not protruding ventrally; anterior tentorial pits not visible; lower face with short, radiatingly strigose sculpture, crests originating from sides of clypeus and reaching less than one‑third of the lower face height; the lateralmost carinula longer than the others (Fig. 2B). Face punctured sublaterally and laterally; malar sulcus a faint, but complete groove (Fig. 2B). Lateral outline of gena hardly convex frontally; gena ventrally carinate, entirely punctured behind sulcus (Fig. 4A). Raised postgenal lamina present, but postgenal depression absent.

Antenna. Scape linear, with five funiculars; all setae subdecumbent (Fig. 2D). Clavomeres fused. Relative length (width) of scape 60.9 (15.0), pedicel 15.7 (12.6), anellus 4.4 (10.0), F1 to F5: 33.8 (19.6), 27.8 (18.9), 27.0 (19.0), 24.3 (19.4), 23.6 (19.6); clava 48.0 (18.1).

Mesosoma 1.7× as long as wide and 1.3× as long as height. Pronotum and mesoscutum 4× and 1.9× as wide as long, respectively; mesoscutellum 1.3× as long as wide. Dorsal surface of mesosoma nearly regularly arched (Fig. 3A); propodeum gently sloping, forming an angle of approximately 50~60° with main axis of mesosoma (Fig. 3B). Pronotum and mesonotum densely punctured with narrow interspaces (Fig. 3A); setation subdecumbent, the setae longer than diameter of punctures on pronotum and on mesoscutellum. Sides of pronotum slightly convex; lateral panel with deep longitudinal depression delimited by distinct ridges (Fig. 3B). Notauli distinctly impressed. Axillar grooves separated by a shorter distance on trans-scutal line than notauli, deeply impressed posteriorly, but vestigial anteriorly; hind margin of mesoscutellum regularly arched (Fig. 3A). Postscutellum with inverted V‑like raised stripe. Propodeum with incompletely delimited, areolate rugose, flat median groove; surface regularly areolate outside the groove (Fig. 3A). Lateral panel of prepectus with rugulae ventrally; deep pit present below the panel; ventral prepectal belt with sharp median tooth (Fig. 3B). Epicnemial carina complete and entirely delimiting the epicnemium distinctly protruding mesally and elbowed with short ventral shelf (Fig. 3C); adscrobal surface of mesepisternum and mesepimeron areolate and bearing long setae; femoral depression variously areolate, rugose ventrally; lower and upper mesepimeron both areolate. Metepimeron coarsely areolate and bearing long lanceolate setae (Fig. 3B).

Legs. Procoxa with deep oblique depression laterally delimited by raised carina, hence separating front surface from side (Fig. 2E). Mesocoxa without lamella. Metacoxa bare basidorsally.

Fore-wing with white setae, 2.4× as long as wide, cc:mv:pmv:stv = 7.7:1.0:2.3:1.3. Membrane of wing with short setation everywhere (Fig. 4B).

Metasoma. Petiole longer than wide, with reticulate dorsal surface, lateral denticles vestigial (Fig. 4C). Gaster lanceolate, about as wide as and 1.5× as long as mesosoma, 2.4× as long as high (Fig. 3D). Gt4 1.4× as long as Gt3 on median line. Gt2–Gt6 reticulate laterally, Gt3–Gt5 with short subdorsal lines of setae; Gt5 and Gt6 entirely, but sparsely setose. Syntergum short and upturned. Apex of ovipositor sheaths sharp.

**Male**. Body length 2.2 mm. Colour similar to female, except antenna entirely dark brown to black (Fig. 1C and D). Scape with distinct apicoventral swelling (ventral plaque). Flagellum with five funiculars, each distinctly pectinate, bearing two rows of long, white, erect setae that exceed segment width and one row of irregularly arranged multiporous plate sensilla (Fig. 4D). Clava fused. Otherwise as in female.

#### Diagnosis

Mesocoxa apparently without lamella (Fig. 2E); lower face with radiating carinulae, originating from sides of clypeus and reaching less than one‑third of the lower face height; frons with faint pre-orbital ridge, antennal scrobes completely delimited by carinate (Fig. 2B); scape yellow; clava apparently fused (Fig. 2D); ventral shelf of mesopleuron angulate with epicnemium (Fig. 3B); propodeum and petiole typical of *Aximopsis* (Fig. 3A, D and Fig. 4C); fore-wing with white setation; marginal vein not longer than the stigmal (Fig. 4B); legs, except coxae, broadly reddish, gaster lanceolate (Fig. 1A). This species is similar to *A. nodularis* (Figs 5, 6), but can be separated by the colour of the antenna (all black in *A. nodularis* vs. scape and pedicel and first flagellomere yellow in *A. qiong*); ratio of mv:pmv (subequal in *A. nodularis* vs. pmv > 2× that of m in *A. qiong*); hair on the mesepimeron (absent in *A. nodularis* vs. present in *A. qiong*).

#### Etymology

The specific epithet “qiong” is an arbitrary combination of letters derived from the Chinese abbreviation “Qiong” (琼), referring to Hainan Province. It is to be treated as a noun in apposition.

#### Distribution

China (Hainan).

#### Biology

This species is a gregarious ectoparasitoid (Fig. 7) reared from trap nests of *Ceratina smaragdula* (Hymenoptera, Apidae) collected in Wenchang and Yongxing Island, Hainan, China. Several host larvae within a single nest tube were parasitised. Based on laboratory rearings from Wenchang, 71 females and 20 males were obtained from 12 parasitised nests, with an overall female:male ratio of 3.6:1. Individual parasitised hosts usually bore 1-5 parasitoids. Parasitoids emerged in late May from Yongxing Island of Sansha City and in August from Wenchang Town of Wenchang City.

#### Variation

The body length of female ranges from 4.2 to 5.2 mm, the body length of male ranges from 2.2 to 3.1 mm. Notauli variable, from weakly to distinctly impressed.

##### Molecular result

The newly-generated *COI* sequences after trimming resulted in a 675 bp matrix, with GTR+F+I+G4 selected as the best evolutionary model, selected by ModelFinder. The tree did not recover *Aximopsis* as a monophyletic clade, instead being repeatedly rendered paraphyletic by *Chryseida*. *Aximopsis qiong* was recovered as sister group to *A. balajasi* and *A. collina*, albeit with low UFboot support (Fig. 8).

## Discussion

This study reports *Aximopsis qiong* sp. nov., a new species of eurytomid parasitoid reared from larvae of the small carpenter bee *C. smaragdula* in Hainan, China. From a taxonomic perspective, *A. qiong* represents the first record of the genus *Aximopsis* from China and only the second species recorded in association with *Ceratina* nests, following *A. nodularis* (UCD Community 2023). The sister-group placement of *A. qiong* with other members of the Palaearctic *nodularis* group is only weakly supported by *COI* data, underscoring the need for a comprehensive taxonomic revision of *Aximopsis* *sensu lato* to clarify phylogenetic relationships amongst the cosmopolitan *Aximopsis* and the two New World genera *Axima* and *Chryseida*.

Beyond its taxonomic significance, the discovery of *A. qiong* carries important ecological and management implications, given the role of its host. *Ceratina smaragdula* is a widely distributed pollinator across southern Asia and plays a significant role in sustaining both natural plant communities and agricultural productivity through its pollination services (Ali et al. 2024). On Hainan Island and its associated archipelagos, this bee represents a particularly important wild pollinator in coastal ecosystems, where it extensively utilises dead twigs of the native littoral shrub *S. taccada* for nesting (unpublished data). Our field surveys reveal that this key pollinator is subject to significant parasitoid pressure from *A. qiong*. This gregarious ectoparasitoid exhibits a strongly female-biased sex ratio (3.6♀:1♂), a trait that would be expected to exacerbate its negative impact on the reproductive success of local *C. smaragdula* populations.

These findings raise a critical concern for pollinator management. Artificial trap nests constructed from native plant materials (like *S. taccada* twigs in this study) are increasingly recognised as an effective strategy for monitoring and augmenting solitary bee populations to enhance pollination services (Rahimi et al. 2021). Our results confirm that *C. smaragdula* readily accepts such nests, making it a promising candidate for managed population enhancement in island and coastal restoration projects (unpublished data). Nevertheless, without appropriate precautions, trap nests may inadvertently function as concentrated breeding resources for *A. qiong*, facilitating its local population increase and intensifying parasitism pressure on the targeted pollinator. Under such conditions, trap-nesting initiatives risk undermining or even reversing their intended conservation outcomes (MacIvor and Packer 2015). To prevent the inadvertent introduction and amplification of *A. qiong* in managed bee populations, we recommend that trap-nesting programmes implement routine inspections and, where feasible, remove or destroy parasitised brood cells prior to parasitoid emergence.

Notably, *C. smaragdula* has recently been documented as an invasive species in Hawai'i (Shell and Rehan 2017). While this invasive context might invite speculation about the potential use of of *A. qiong* for biological control, the lack of host specificity documented in congeners, such as *A. nodularis*, makes such application highly uncertain. More importantly, the primary value of documenting *A. qiong* in its native range lies in the warning it provides for local conservation: in Hainan, where *C. smaragdula* serves as a key pollinator, trap‑nesting programmes could inadvertently spread this parasitoid and undermine pollination services. Management efforts should, therefore, prioritise excluding *A. qiong* from augmented *C. smaragdula* populations, rather than promoting it as a biocontrol agent.

## Supplementary Material

XML Treatment for Aximopsis qiong

## Figures and Tables

**Figure 1.**
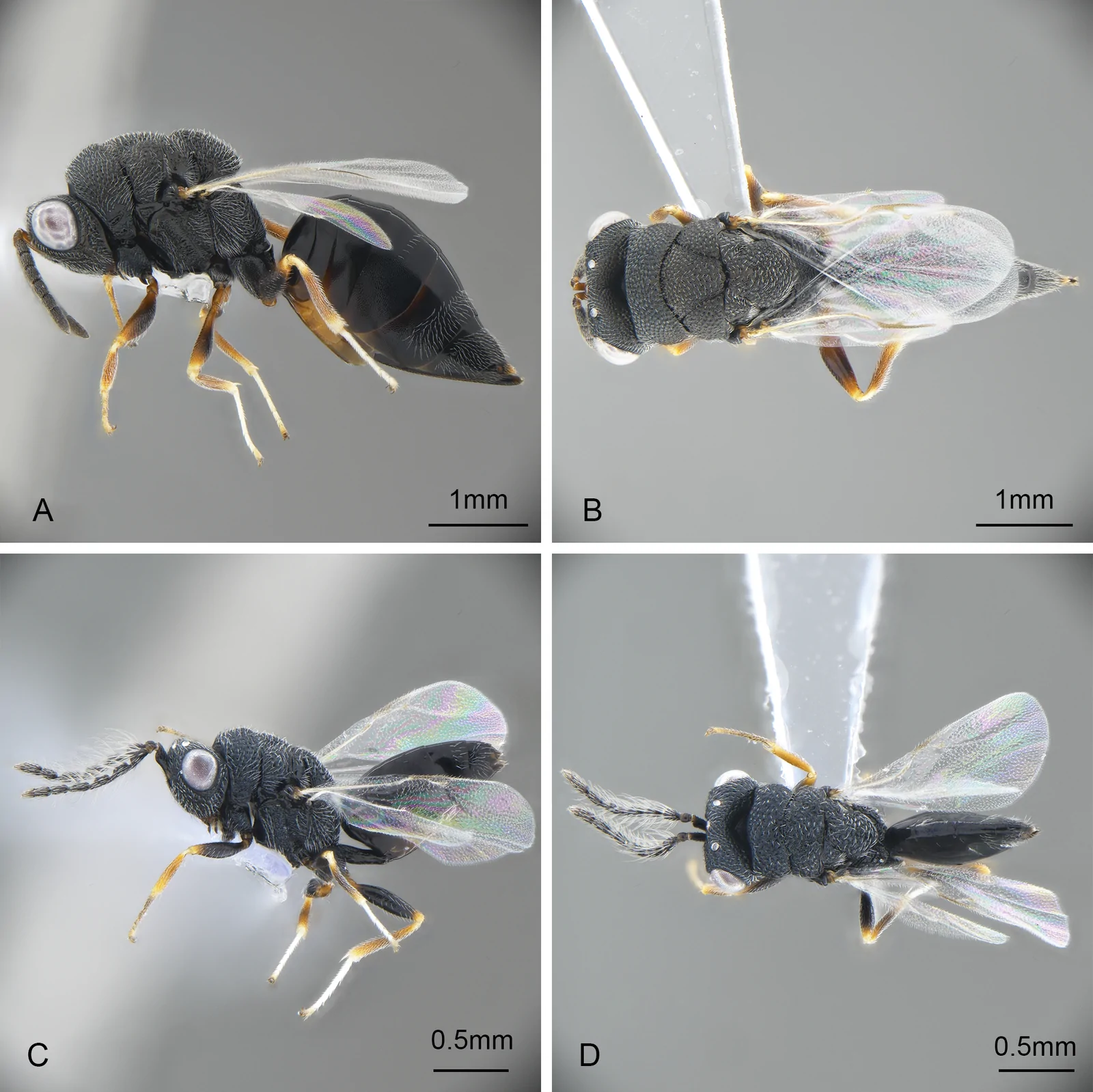
*Aximopsis qiong* sp. nov. holotype, female (voucher E0016503): **A** lateral habitus; **B** dorsal habitus. paratype, male (voucher E0015708): **C** lateral habitus; **D** dorsal habitus.

**Figure 2.**
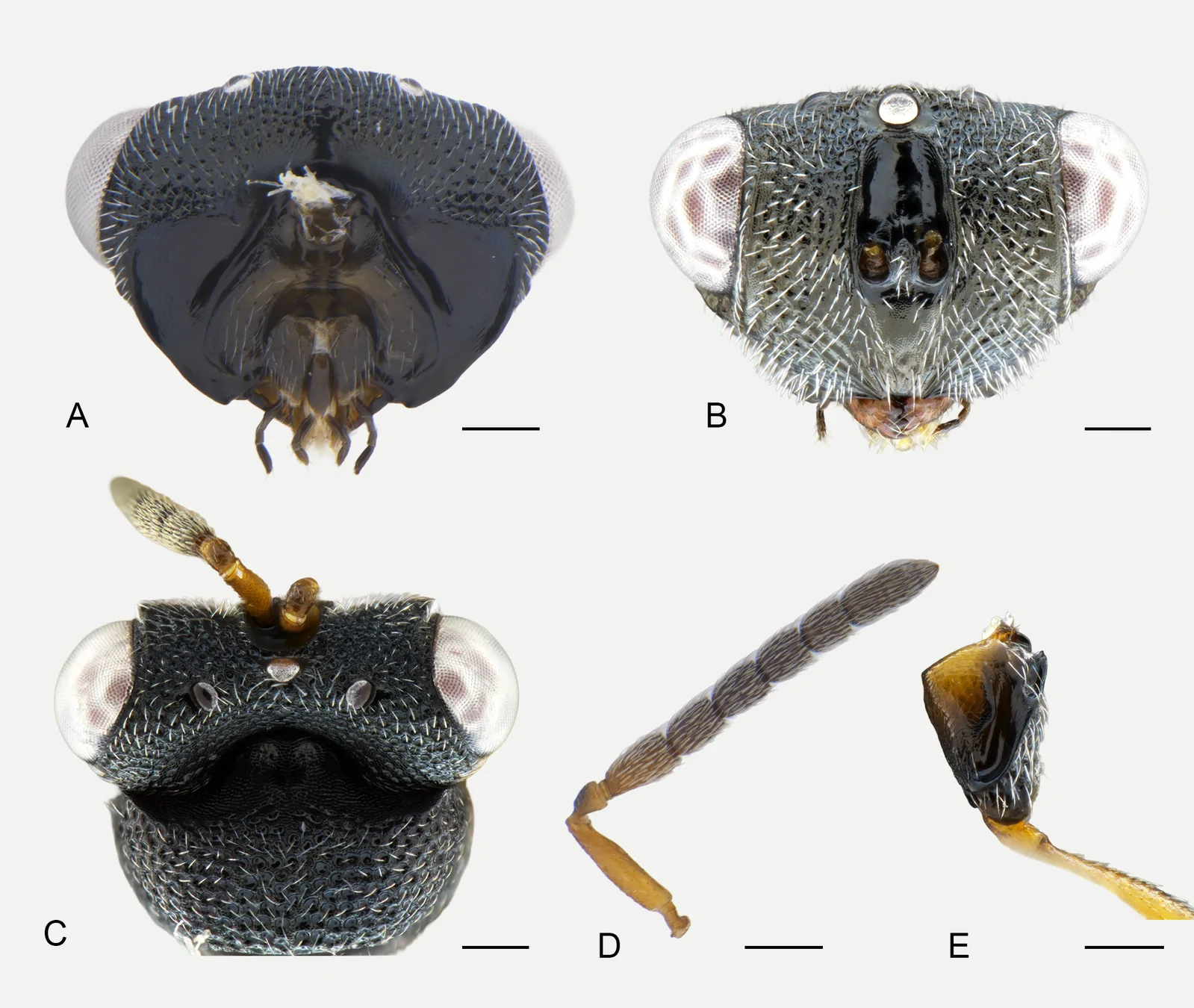
*Aximopsis qiong* sp. nov. female: **A** head, posterior view; **B** head, frontal view; **C** head, dorsal view; **D** antenna; **E** procoxa, anterior view. Scale bars = 0.2 mm.

**Figure 3.**
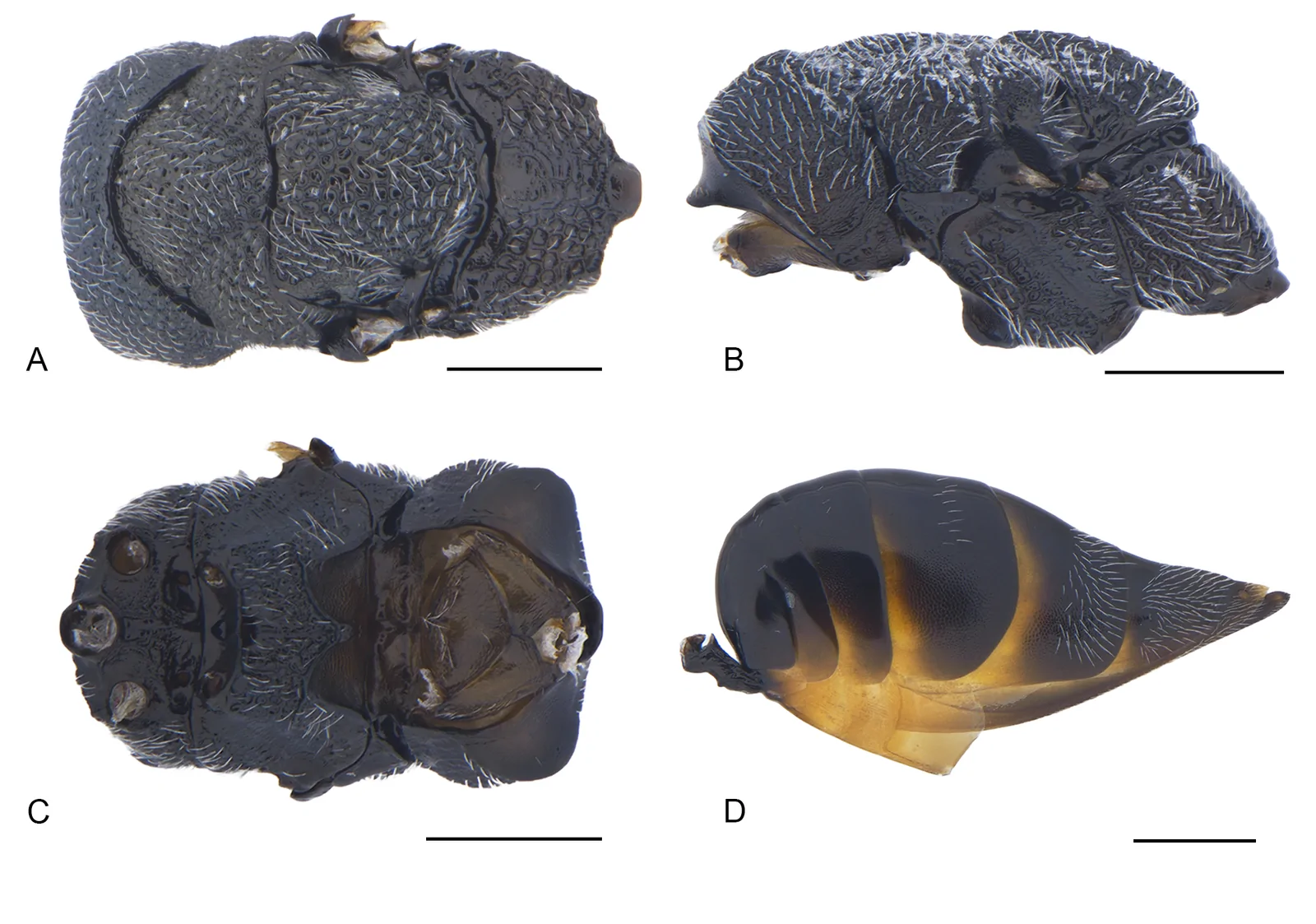
*Aximopsis qiong* sp. nov. Female: **A** mesosoma, dorsal view; **B** mesosoma, lateral view; **C** mesosoma, ventral view; **D** metasoma, lateral view. Scale bars = 0.5 mm.

**Figure 4.**
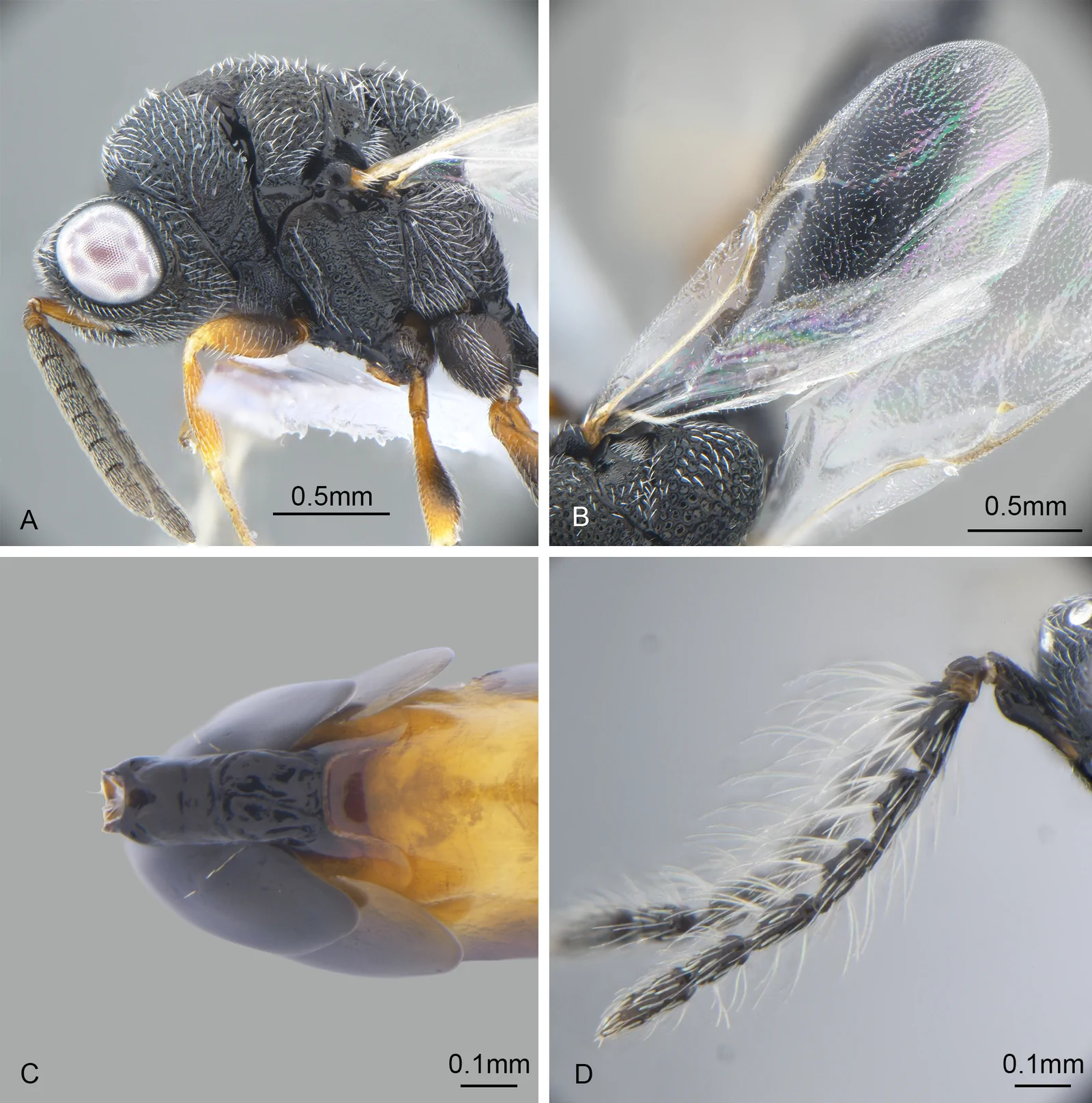
*Aximopsis qiong* sp. nov. paratype, female (voucher E0015707): **A** head, lateral view; **B** fore-wing; **C** petiole, ventral view. paratype, male (voucher E0015708); **D** antenna.

**Figure 5.**
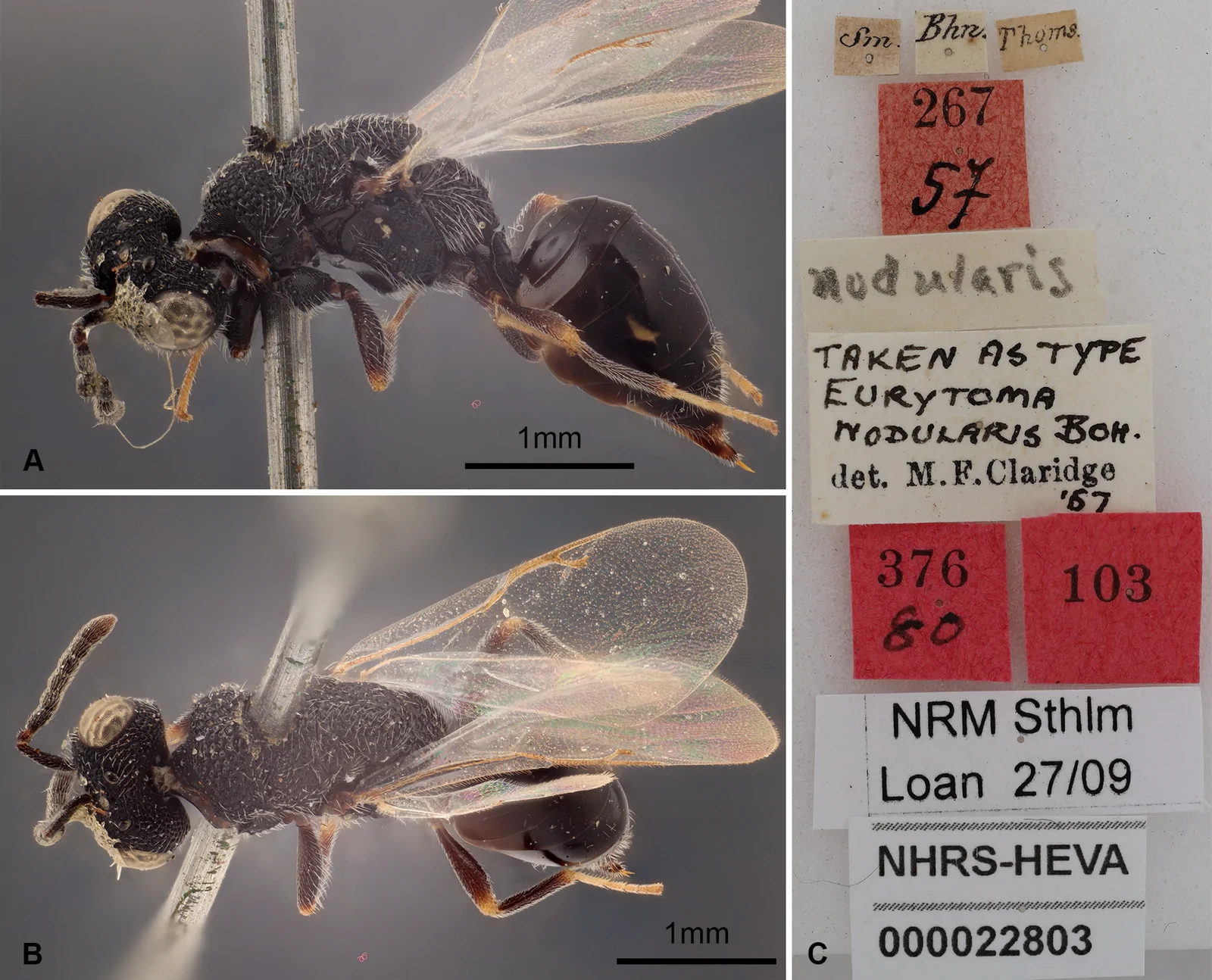
*Aximopsis nodularis* holotype (SMNH, NHRS-HEVA-000022803), female: **A** lateral habitus; **B** dorsal habitus; **C** labels. Photos by Hege Vårdal.

**Figure 6.**
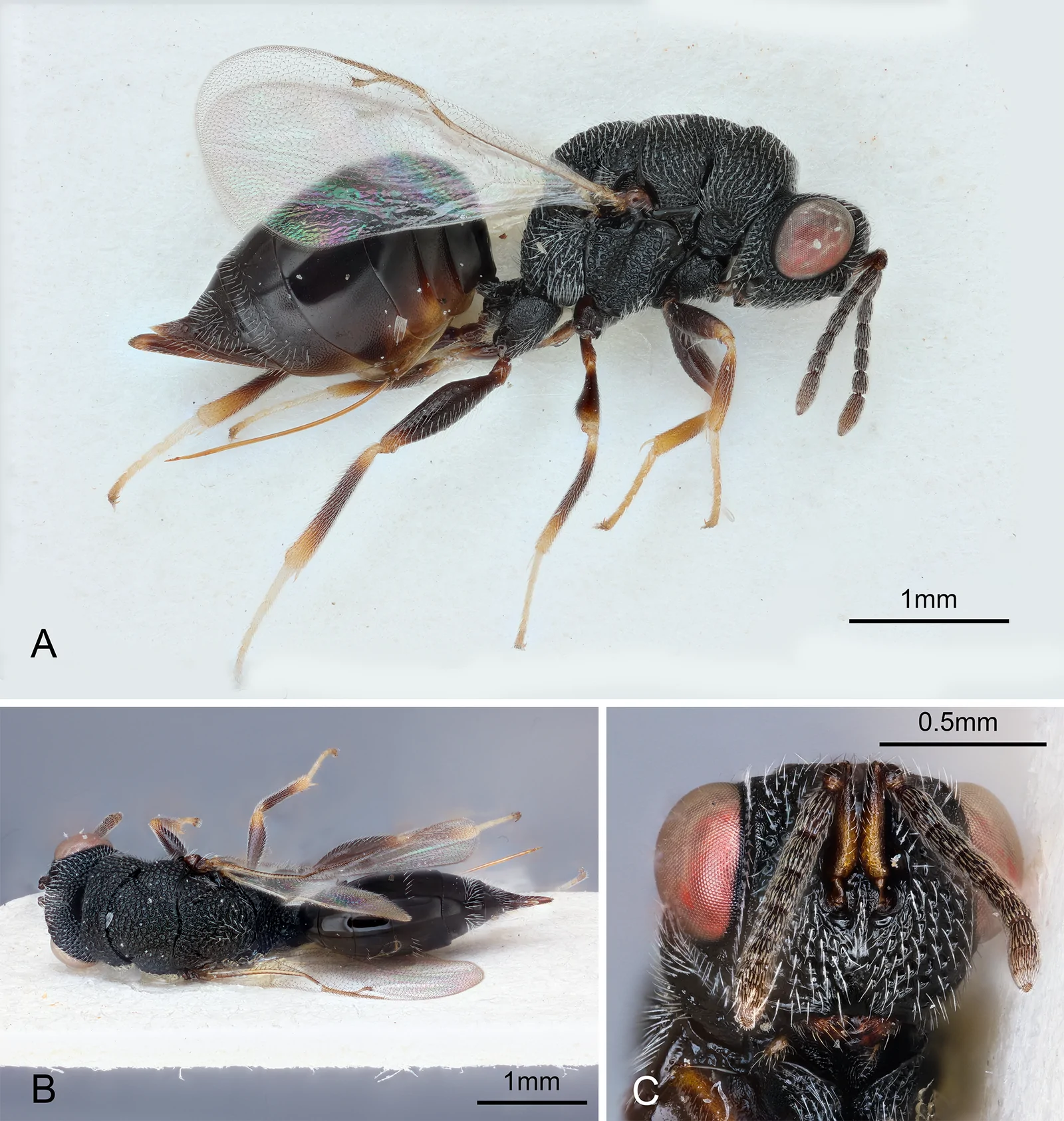
*Aximopsis nodularis* (NMNH, USNMENT01570805), female: **A** lateral habitus; **B** dorsal habitus; **C** head, frontal view. Photos by R. Luke Kresslein.

**Figure 7.**
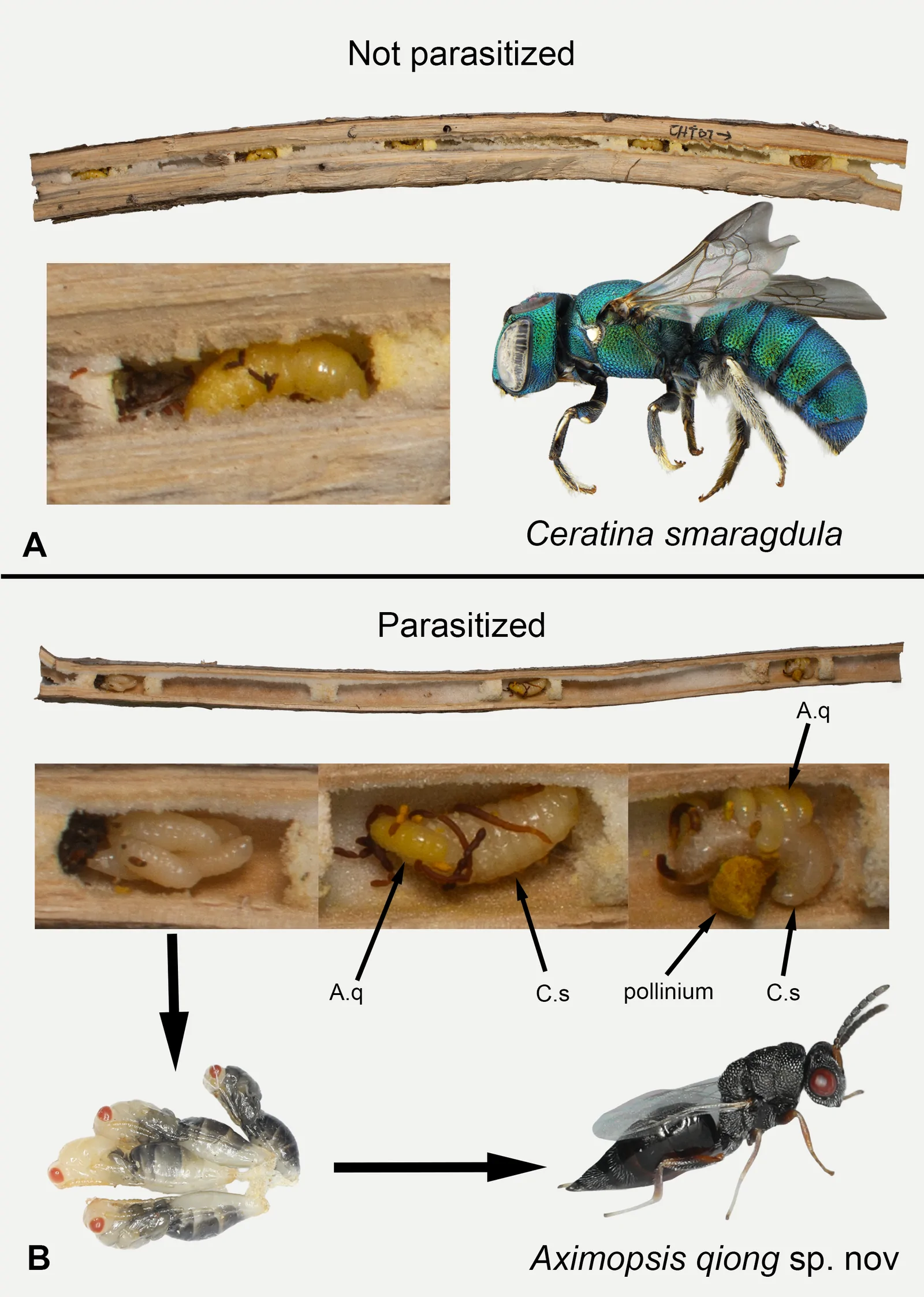
Biological aspects of *Aximopsis qiong* sp. nov.: **A** Cross section of *Scaevola taccada* twig showing a non-parasitised larva of *Ceratina smaragdula* (top), close up of the larva (bottom left) and an adult (bottom right); **B** Cross section of the *Scaevola taccada* twig showing the parasitised larvae of *Ceratina smaragdula* in different stages of attack by *Aximopsis qiong* (top), mature pupae of *A. qiong* (bottom left) adult female of *A. qiong* (bottom right).

**Figure 8.**
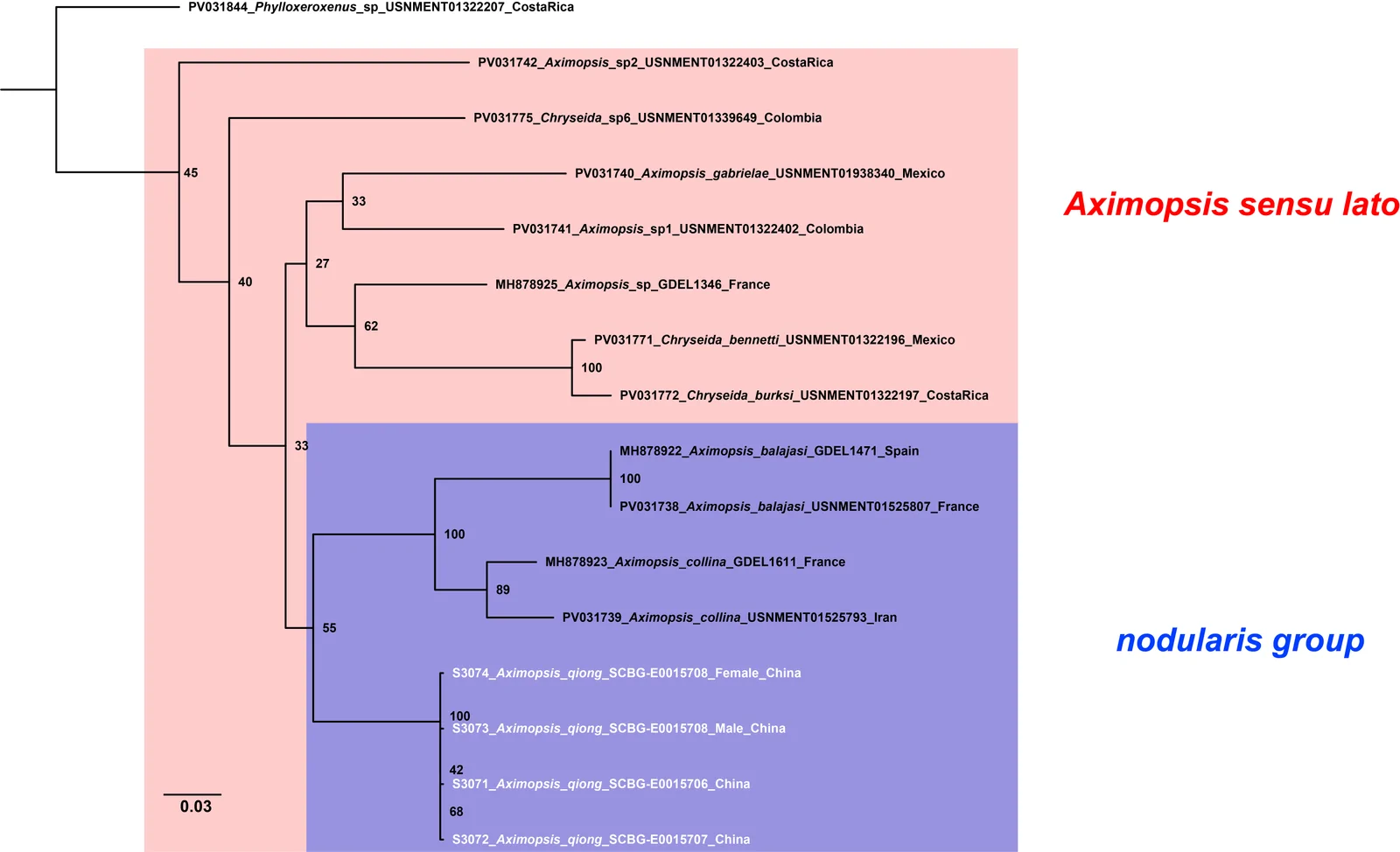
Maximum Likelihood tree of *Aximopsis* *sensu lato* inferred from *COI* gene sequences. Numbers at the nodes indicate ultrafast bootstrap values.
